# DIVERSE CONTEXTS, UNIQUE EXPERIENCES: IMPACT OF SOCIAL ISOLATION ON PSYCHOSOCIAL FACTORS AND COGNITION

**DOI:** 10.1093/geroni/igac059.121

**Published:** 2022-12-20

**Authors:** Jess Francis

## Abstract

The aging population is not a monolithic group. Older adults’ experiences of and behaviors within their social networks are as varied as their diverse backgrounds and lifestyles. Although the threat of social isolation is particularly salient among older adults, the psychosocial and cognitive impact can differ along demographic and socioeconomic lines. The objective of this symposium is to highlight how different older adults are effected by their social network characteristics and structure, and how they mobilize their psychosocial resources to protect against loneliness, depression, and threats to cognition. First, Hong & Mejia examine how older men and women compare in their use of information and communication technology (ICT) as each influenced the experience of loneliness during the COVID-19 pandemic. Francis & Brauer also look at ICT use as they assess demographic and socioeconomic differences in social isolation, perceived mattering, and Facebook use among single older adults who are childless and live alone. Oh & Chopik explore uncoupled older adults along demographic lines in a 10-year longitudinal study of single people, their perceptions of change, satisfaction with singlehood, and major life events. Further exploring the theme of well-being during the COVID-19 pandemic, Cooper, Ajrouch, & Antonucci assess cognition, depressive symptoms, and their association with race among a sample of Whites, Blacks, and Arab Americans. Finally, Lee & Colleagues round out this symposium by sharing results of their study in which they examine the coping styles of non-Hispanic Whites and non-Hispanic Blacks and the impact on cognition.

